# Age, Sex, and Telomere Dynamics in a Long-Lived Seabird with Male-Biased Parental Care

**DOI:** 10.1371/journal.pone.0074931

**Published:** 2013-09-04

**Authors:** Rebecca C. Young, Alexander S. Kitaysky, Mark F. Haussmann, Sebastien Descamps, Rachael A. Orben, Kyle H. Elliott, Anthony J. Gaston

**Affiliations:** 1 Department of Biology and Wildlife, Institute of Arctic Biology, University of Alaska Fairbanks, Fairbanks, Alaska, United States of America; 2 Biology Department, Bucknell University; Lewisburg, Pennyslvania, United States of America; 3 Norwegian Polar Institute, Fram Centre, Tromsø, Norway; 4 Department of Ocean Sciences, Long Marine Laboratory, University of California Santa Cruz, Santa Cruz, California, United States of America; 5 Department of Biological Sciences, University of Manitoba, Winnipeg, Manitoba, Canada; CNRS, Université de Bourgogne, France

## Abstract

The examination of telomere dynamics is a recent technique in ecology for assessing physiological state and age-related traits from individuals of unknown age. Telomeres shorten with age in most species and are expected to reflect physiological state, reproductive investment, and chronological age. Loss of telomere length is used as an indicator of biological aging, as this detrimental deterioration is associated with lowered survival. Lifespan dimorphism and more rapid senescence in the larger, shorter-lived sex are predicted in species with sexual size dimorphism, however, little is known about the effects of behavioral dimorphism on senescence and life history traits in species with sexual monomorphism. Here we compare telomere dynamics of thick-billed murres ( *Uria* *lomvia* ), a species with male-biased parental care, in two ways: 1) cross-sectionally in birds of known-age (0-28 years) from one colony and 2) longitudinally in birds from four colonies. Telomere dynamics are compared using three measures: the telomere restriction fragment (TRF), a lower window of TRF (TOE), and qPCR. All showed age-related shortening of telomeres, but the TRF measure also indicated that adult female murres have shorter telomere length than adult males, consistent with sex-specific patterns of ageing. Adult males had longer telomeres than adult females on all colonies examined, but chick telomere length did not differ by sex. Additionally, inter-annual telomere changes may be related to environmental conditions; birds from a potentially low quality colony lost telomeres, while those at more hospitable colonies maintained telomere length. We conclude that sex-specific patterns of telomere loss exist in the sexually monomorphic thick-billed murre but are likely to occur between fledging and recruitment. Longer telomeres in males may be related to their homogamous sex chromosomes (ZZ) or to selection for longer life in the care-giving sex. Environmental conditions appeared to be the primary drivers of annual changes in adult birds.

## Introduction

Individuals vary in their rate of senescence, measured by declines in physiological and reproductive performance [1]. Sex, with its suite of genetic, physiological, and behavioral effects, is an obvious source of this variation. Sex-specific life histories may be driven by many factors: long life in either gender can be driven by differential parental investment [2]; low growth rates [3]; and sex-specific genetic causes, including estrogen or homogamy (in mammals, females are XX and may inactivate a chromosome, this is reversed in birds: males are ZZ while females are ZW) [4]. Mammalian females usually live longer than males despite providing most offspring care. However, females also possess life-extending traits: slower growth rates, smaller adult sizes, higher estrogen, and homogamy [4], which may counterbalance the costs of high parental investment. Avian systems may help disentangle these mechanisms because the homogametic sex is the male, separating effects of parental investment from some genetic and endocrine causes (e.g. [5]). This study addresses telomere decay rates in a structurally monomorphic seabird where parental care is provided by both sexes for the first two to four weeks, and exclusively by males for one to two months post-fledging [6].

Senescence effects on survival rates or reproductive output are often catastrophic, developing quickly shortly before death [7]. However, individual-specific deterioration of body reserves may not be well predicted by chronological age [8]. We refer to chronology-decoupled aging as biological aging and measure it with telomere length (TL). TL may reflect aging on a physiological and cellular level, as well as incorporating chronological age and usually shows steady rates of decline instead of catastrophic loss, making it an excellent candidate for a biomarker of individual biological age [8]. TL is measured in a variety of ways in the ecological literature. The most common methods are the Southern blot-derived telomere restriction fragment (TRF) assay [9–11], smaller windows of the TRF smear [12], and quantitative PCR [8,13,14]. Here we compare the effects of age, sex, and colony on telomere length as estimated by each of those methods.

Sex-specific TL is documented in many taxa [15,16], but often in species with female-biased parental care and large sexual dimorphism in body size. When neonates do not differ in TL, such differences may be related to level of parental investment or developmental rates [3]. However, it is possible that telomeres would be more protected in the homogametic gender, which can select between two germ-line sex chromosomes [5,17], or the gender which receives more parental investment in the nest [18]. Little work has focused on systems with morphologically uniform sexes but behavioral differences in parental care.

The thick-billed murre ( *Uria* *lomvia* ) is a long-lived seabird in the Alcidae family. Like other alcids, it has slow maturation, low annual fecundity (obligate clutch of one), and high adult survival [6]. Thick-billed murres are monogamous birds which share incubation and nestling care (2-4 weeks) equally [6], and the sexes are essentially monomorphic. Nonetheless, strong behavioral dimorphism manifests in male care; males provide several months of post-fledging care to the chick, which leaves the colony at only 1/3 of adult size. Females are not associated with offspring after fledging [6]. Here we examine the relationship between chronological age and telomere length in thick-billed murres both cross-sectionally and longitudinally. Cross-sectional sampling allows for an assessment of chronological age effects on TL, but does not eliminate the effects of selective disappearance or cohort differences on the emerging patterns. However, longitudinal sampling, even in birds of unknown age, addresses the mechanisms of aging – potentially eliminating selective disappearance or cohort effects as drivers of the patterns seen in cross-sectional studies [19]. Telomere dynamics are expected to differ by gender when the sexes are very differently sized (not applicable to murres), reproductive investment differs strongly, or due to genetic and evolutionary differences in protective mechanisms. These latter two effects could drive differences in telomere losses between male and female murres. We predict that males will show faster rates of biological aging (determined by telomere attrition) due to heavier reproductive investment. This would result in adult males having lower TL than females, and a more negative slope in the cross-sectional sampling. Longitudinally, they should show higher rates of annual TL loss. Our alternative prediction is that males will have slower telomere attrition due to the benefits of homogamy, resulting in higher TL than in females, shallower cross-sectional slope, and slower rates of loss. Lastly, since conditions affecting telomere attrition or ability of murres to regulate telomere changes may differ by colony, and murres are highly faithful to their breeding sites, we included colony as an explanatory factor in the longitudinal analysis.

## Materials and Methods

### Ethics Statement

This study was approved by the University of Alaska Fairbanks IACUC (156937-3 to A. S. Kitaysky), Canadian Wildlife Service NWRC & Ontario Region Animal Care Committee (0800AG01 to A. J. Gaston), and the Canadian Wildlife Service (NUN-SCI-08-55 to A. J. Gaston and NUN-SCI-6-01 to M. Mallory). All sampling was done under approved guidelines of these agencies. All field work was carried out under appropriate regional and federal permits. Collection at St. Paul Island (57°08’ N, 170°18’ W), St. George Island (56°36’ N, 169°39’ W), and Bogoslof Island (53°56’ N, 168°02’ W) was authorized by the US Fish and Wildlife Service and the Alaska Maritime National Wildlife Refuge. Collection at Coats Island (62°57′N, 82° 00′W) was authorized by the Kivalliq Inuit Association, and collection at Diabasodden (78°21’ N, 16°8’ E) was authorized by the Governor of Svalbard (program number 361).

### Techniques and Sample Collection

Currently, several methods are used to measure telomeres in the fields of ecology and evolution [20]. Thus, to model the relationship between telomeres and chronological age, we used two assay techniques, yielding three measures of telomere length or quantity. The first technique, from which two measurements are derived, was the telomere restriction fragment assay, a modified Southern blot visualizing terminal telomere repeat densities at all molecular weights, measuring all cells, all chromosomes, and all telomere lengths. The first measure (which we call TRF) is the average telomere length in the entire smear. The second measure, derived from the same TRF smear, was the telomere optimal estimate (TOE), which measures only the lower molecular weight region of the TRF smear: our upper cut-off was 5 kb for TOE, the lower cut-off was the same as for TRF and followed Salomons et al. [10]. This measure may be more sensitive to telomere shortening, as it focuses on the shortest telomeres in the distribution [12]. TRF and TOE were calculated according to Salomons et al. [10] and Haussmann and Mauck [12]. Briefly, whole blood, stored frozen in a glycerol buffer, was extracted into agarose plugs using a kit (Chef Genomic DNA Plug Kit, Bio-Rad) and digested with a mixture of 3 U HinfI, 15 U HaeIII, and 40 U RsaI (Roche Applied Science). Plugs were separated using pulsed field gel electrophoresis (Bio-Rad ChefMapper) on a 0.8% agarose gel. Run parameters were 21 hours at 3 V/cm and 0.5-7s switch times. The buffer (0.5X TBE) was circulated and kept at 14 °C. Gels were hybridized at 37 °C with 3,000,000 cpm of the telomere-specific radio-labeled oligo (CCCTAA)_4_. After hybridization, rinsing and visualization followed Haussmann and Mauck [12]. Samples were analyzed in random order on four gels, and control samples were run twice in each gel to determine inter- (12.8% - TRF; 3.2% - TOE) and intra-assay variability (< 5% per gel).

The second technique, and third measure, quantitative PCR (qPCR) produced a ratio of the quantity of telomeric DNA to a reference gene (T/S ratio), but did not provide lengths. This technique unavoidably includes interstitial repeats (those internal to the chromosome and unaffected by aging). Protocols were adapted from Cawthon [21], reference gene primers were from Criscuolo et al. [14], and telomere primers were from Foote [22]. Samples were run on one plate for telomere primers and another for the reference gene primers, and in each plate samples were in triplicate. Samples where the coefficient of variation was > 10% were excluded from analysis. Efficiencies of amplification were 100% and 108% for the reference gene and for telomeres, respectively.

Known-age individuals (Table 1) were sampled on Coats Island, Nunavut, Canada. Adults were not sampled longitudinally on this colony. Chicks (< 14 days old) were sampled from several colonies: Coats Island; Diabasodden on Svalbard; Bogoslof Island, in the Aleutian Chain; and St. Paul Island in the central Bering Sea. Sample sizes between analysis methods differ; we prioritized the TRF assay, of which TOE is a derived value, which did not always leave sufficient sample for a concentrated high-quality DNA extract for qPCR. Longitudinal murre sampling (Table 2) was carried out on Diabasodden, Bogoslof Island, St. Paul Island, and St. George Island (also central Bering Sea). Birds were captured during the breeding season: late July and early August. Recaptures were done in the following breeding season. Most birds (n = 67) were recaptured after one year. Two individuals were recaptured after two years, and their change in telomere length was divided in half to reflect changes in a “per year” format. Blood (0.2-1 mL) was collected from the brachial vein. Sampling was not lethal and handling time minimized. Longitudinal sampling was analyzed using TRF and TOE and the changes to these measures (ΔTRF and ΔTOE); there were not enough red blood cells remaining to conduct qPCR analysis. Birds were genetically sexed following Griffiths et al. [23].

**Table 1:** Sample sizes of cross-sectionally sampled birds. All adults were sampled on Coats Is. while chicks came from four colonies (see Methods for details).

Technique		TRF & TOE	qPCR
Adults	Males	38	30
	Females	9	5
Chicks	Males	9	4
	Females	4	2
All Ages	Both Sexes	60	41

**Table 2:** Sample sizes and colony of origin for longitudinally sampled birds. All birds were breeding adults of unknown chronological ages.

Colony	Bogoslof	St. George	St. Paul	Diabasodden	All Colonies
Males	8	9	10	10	37
Females	6	4	11	11	32
Total	14	13	21	21	69

### Statistics

**Figure 1:**
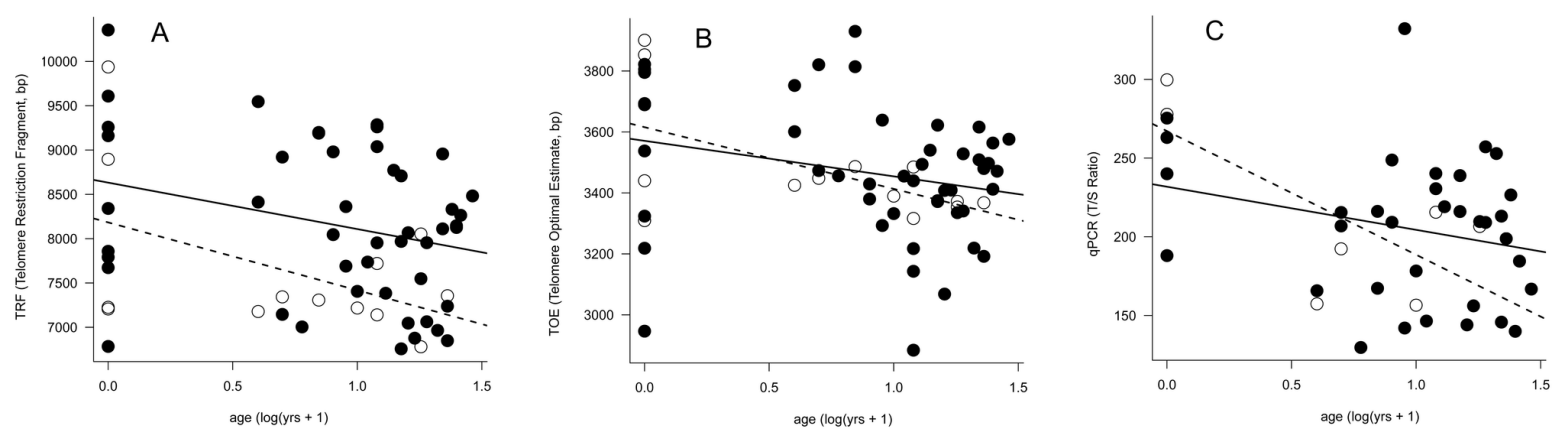
Telomere loss with age and sex in thick-billed murres. Telomere length is measured as TRF (A), TOE (B), and qPCR (C). Males are closed circles and solid lines. Females are open circles and dashed lines.

All statistics were done in the Program R, vs. 2.12.2 [24]. Results are presented as mean and 95% confidence intervals. Where necessary, variables were transformed to meet parametric assumptions. The contribution of chronological age and sex to each of three TL measures (TRF, TOE, and qPCR) was modeled with ANCOVA, which included age, sex, and the age x sex interaction term. Despite the appearance of more variability in male telomere lengths (Figure 1), variability by gender was not significantly different (TRF: Bartlett’s K^2^ = 0.0077, p = 0.93; TOE: Bartlett’s K^2^ = 0.64, p = 0.43; and qPCR: Bartlett’s K^2^ = 0.36, p = 0.54). Most telomeres are lost during periods of rapid growth, in seabirds between hatching and recruitment [25], therefore the predicted shape of the relationship with age is non-linear. To allow linear modeling, age was transformed as log(age+1). This transformation allowed for inclusion of chicks, which otherwise would be excluded as log(0). For longitudinal sampling, which was only available for adults, simple two-way ANOVAs were used to assess the effect of sex and colony on TRF, TOE, and the percent change in TRF and TOE between 2 years in the same individual. When comparing telomere lengths for longitudinally sampled birds, multiple samples exist; to control for repeated sampling, a mixed model was used with sex and colony as fixed effects and bird identity as a random effect.

## Results

### Relationships between different measures of telomere length

Quantitative PCR correlations with TRF and TOE approached significance (TRF and qPCR: Pearson’s *r* = 0.29, t = 1.88, p = 0.068; TOE and qPCR, Pearson’s *r* = 0.25, t = 1.60, p = 0.12). TRF and TOE were significantly correlated (Pearson’s *r* = 0.30, t = 2.36, p = 0.022).

### Chronological age and telomeres

Chicks came from different colonies, but colony never affected chick TL, so we have pooled them (TRF: F_3,9_ = 1.92, p = 0.20; TOE: F_3,9_ = 2.86, p = 0.097; qPCR: F_2,3_ = 2.10, p = 0.27) The model for average terminal TL for all cells (TRF) showed significant effects of age (β = -765 bp*year^-1^, F_1,56_ = 5.16, p = 0.027) and sex (β_males_ = 8632 bp, β_females_ = 8183 bp, F_1,56_ = 5.50, p = 0.023), but no interaction (F_1,56_ = 0.212, p = 0.65). TL decreased with chronological age in both sexes, but males had longer TL (Figure 1A). This sex effect was driven by the large difference in adults; chicks did not differ in their TL by sex (t = -0.208, df = 11, p = 0.76), but adult males had higher TRF (t = -2.62, df = 45, p = 0.012) than females. Results for the lower, age-sensitive window (TOE, Figure 1B) were less complex; age had a significant effect (β = -202 bp*year^-1^, F_1,56_ = 5.92, p = 0.018), but sex and the age x sex interaction did not (both p > 0.5). The qPCR model also showed a significant age effect (Figure 1C, β = -78.6T/S* year^-1^, F_1,37_ = 5.63, p = 0.023), a lack of the effect of sex, and no interaction (both p > 0.2).

### Longitudinal sampling

**Figure 2:**
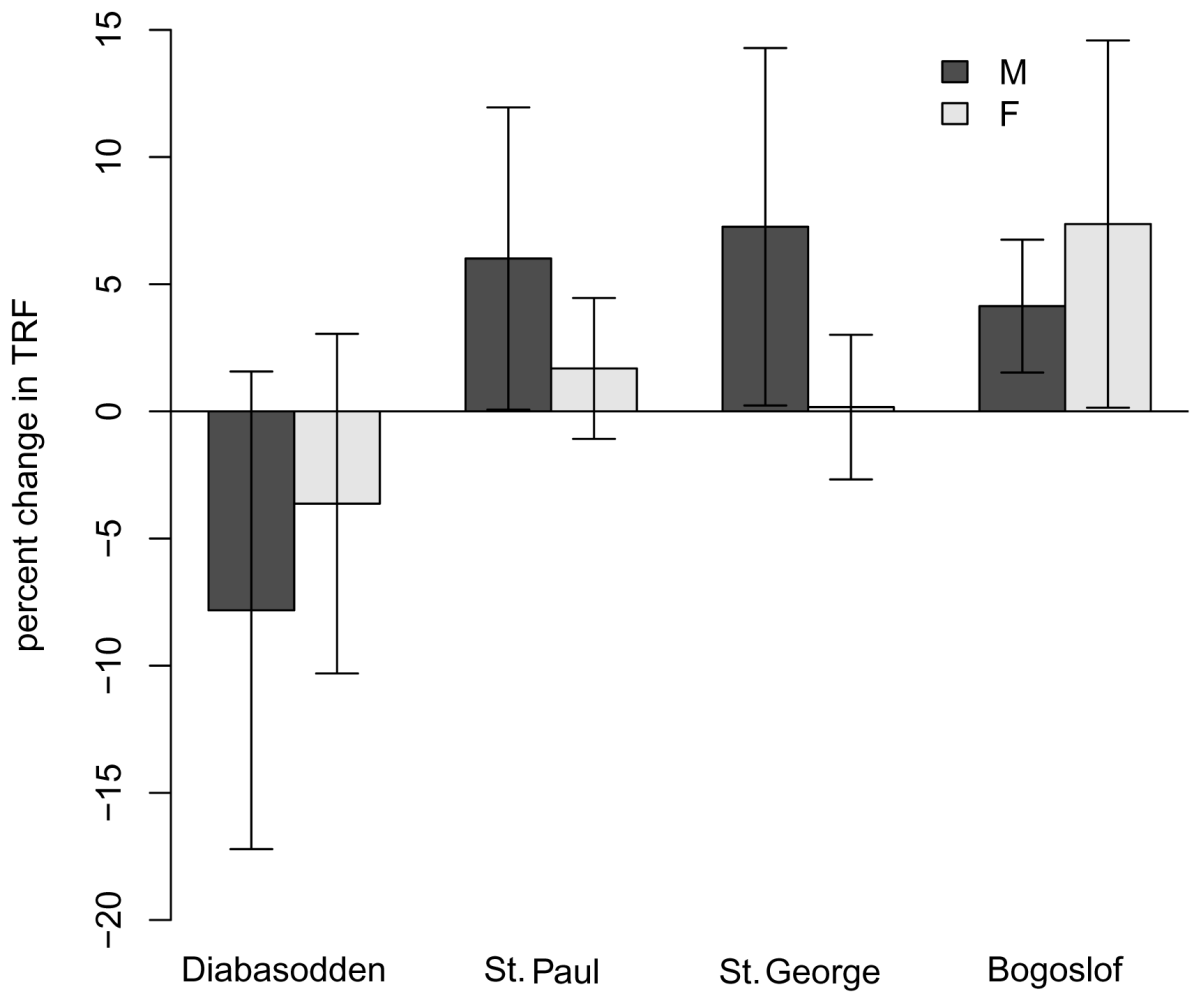
Longitudinal (inter-annual) percent change in telomere length of breeding thick-billed murres depends on colony, but not sex. Diabasodden males lost telomere length, compared to St. Paul or St. George males; all other colonies did not show changes statistically different from zero. At no colony were the differences between the sexes significant. Diabasodden has the poorest conditions and negative population trends and is the only colony where loss occurred. Changes are over one year, and are presented as mean ± 95% confidence interval.

For the four colonies sampled longitudinally, colony (F_3,61_ = 4.41, p = 0.0071) and sex (F_1,61_ = 5.34, p = 0.024) both affected TRF, but there was no interaction (F_3,61_ = 1.58, p = 0.20). Bogoslof had the longest TL, and the other colonies were shorter (Bogoslof: 7805 [CI: 7555-8055] bp; St. George: 7268 [6865-7672] bp; St. Paul: 7044 [6765-7322] bp; Diabasodden: 6865 [6590-7140] bp). Males again had significantly longer TRF than females (males: 7417 [7168-7666] bp; females: 6925 [6752-7098] bp), providing further support to the pattern of higher TRF in males which was also found in the cross-sectional sampling (above). TOE showed a weak effect of colony (F_3,61_ = 2.87, p = 0.044), an effect of sex (F_1,61_ = 5.04, p = 0.028, and a trend for an interaction between the two (F_3,61_ = 2.56, p = 0.064). Interestingly, females had slightly longer TOE than males (males: 3520 [3468-3571] bp, females: 3609 [3567-3652]), but the difference is likely not biologically significant. When comparing the change in TL from the first year of sampling to the second (ΔTRF), there was a strong effect of colony on ΔTRF (Figure 2; F_3,61_ = 4.82, p = 0.0044), but no sex effect or interaction (both p > 0.35). Birds on Diabasodden lost telomeres, while other birds maintained or slightly elongated theirs (Figure 2). This pattern was driven by the loss of telomeres in male Diabasodden birds (Figure 2): Tukey’s post-hoc comparisons showed that most losses or gains did not differ, however, males on Diabasodden had a significantly lower change than males on St. George (p = 0.047) and nearly significantly lower than St. Paul (p = 0.075). ΔTOE showed no effect of sex (F_1,61_ = 2.17, p = 0.14), colony (F_3,61_ = 0.722, p = 0.54), or the interaction (F_3,61_ = 1.09, p = 0.36).

## Discussion

### Comparison of telomere length techniques

This study compared three measures of telomere length or quantity: TRF, TOE, and qPCR. Each parameter quantifies a different subset of telomeric DNA. TRF measures all terminal telomeres; TOE measures all terminal telomeres in a lower window of the smear, and qPCR yields a measure of all terminal and interstitial telomeric DNA relative to a single-copy reference gene. As such, they provide slightly different information on changes in telomere quantities in relation to age and sex of individuals. Each technique showed a significant effect of chronological age on the length of telomeres. However, TRF also showed an effect of sex (Figure 1A). The sexes do not differ when examining only the shorter telomeres (TOE) or when including interstitial repeats (qPCR). Thus, it appears that differences between the sexes may be stronger for long telomeres, which are excluded in the TOE analysis and overwhelmed by the sexually similar interstitial signal in qPCR. TOE is valued for its sensitivity to aging, but in our analysis results were comparable, and including the upper portion of the smear may allow analysis of important factors (e.g. sex) to which TOE did not respond in our analysis.

Reassuringly, qPCR yielded similar results to the TRF and TOE measures of telomere length. Its output was correlated with both TRF and TOE, but its detection of sex effects in conjunction with age may have been diluted by the presence of interstitial repeats [26]. This may also be responsible for the lower correlation between the Southern blot-derived measures, which do not measure interstitial repeats, and qPCR, which does, than have been reported elsewhere (e.g. [14]). It is not uncommon for TRF protocols to denature the DNA before blotting to membrane, which does not occur in our protocol, as we hybridize in the gel itself. Denaturing the DNA allows probing of interstitial repeats as well as terminal repeats, and will increase the correlation between qPCR and TRF measurements. Differences in qPCR output could also be attributable to lower power, due to our smaller qPCR sample size.

### Sex differences in TL in the thick-billed murre

This study presents evidence for sex-specific dynamics of biological aging in a species with male-biased parental care. All measures showed a significant decline in telomeres with chronological age (Figure 1), and the measure of all terminal telomeres (TRF) also was sensitive to an effect of sex, both in the cross-sectional and in the longitudinal sample. Most telomere length is lost from chick to age four (the youngest known-age adult in our sample), and adult telomere length is not strongly driven by chronological age (when chicks are excluded from the analysis, there is no effect of age on telomere length). Lower TL for adult females, found in both the cross-sectional and longitudinal analyses, coupled with no differences in telomere rates of change, implies a larger loss before recruitment in this sex. Good candidates for explaining the variation in TL which remains after accounting for chronological age and sex include hatch-year conditions, physiological stress, and reproductive history [27,28]; such effects on aging would further support the concept of chronologically-independent biological age.

The shorter TL of female murres may have several explanations. The first of these non-mutually exclusive explanations is that inflexible female investment results in a “faster-living” life history, according to the theory of disposable soma. This would result in faster biological aging of females, despite the costly male investment in post-fledging care of young. Females bear high pre-fledging costs, consisting of egg production and post-fledging nest defense [6,29,30]. In addition the metabolic and physiological stress associated with these activities produces oxidative stress, which is known to negatively affect TL [31,32]. The female’s investment is primarily early in the reproductive bout, while male reproductive costs peak post-fledging. If reproduction fails prior to fledging, the female has invested more than the male. Since murre laying and hatching success are relatively high and constant from year to year [33], a female’s reproductive investment is then insensitive to environmental changes; it is constant as long as the egg hatches, but regardless of fledging success. Male investment would then be highest when it has the most payoff and lowest in poor years when conditions do not allow successful reproduction. Common murres ( *Uria* *aalge* ), sister species to the thick-billed murre, show more rapid female senescence and male-biased parental care patterns; this work predicts faster female senescence as the price of high reproductive investment and is consistent with the disposable soma theory [34,35]. The less environmentally sensitive (“risk-prone”) female strategy may reduce survival and result in higher mortality. Lowered survival prospects would select females for a “faster-track” life history involving less somatic investment, including telomere maintenance. If telomere length reflects the evolutionary cost of cumulative reproductive investments [36], then this could explain why females’ telomeres are shorter than those of males, despite occurring primarily before first reproduction.

Other potential explanations for shorter adult female TL is that males with short TL are selectively removed from the population before breeding, however there is no empirical evidence of differential selective forces acting on juvenile murres. Another explanation for short female TL is that murre parents invest more highly in their male offspring. Seabird parents are often willing to invest more energy into male offspring, because the difference between high and low quality males in terms of future reproductive value is much greater than that between high and low quality female offspring [37]. In the common murre, parents provisioning male chicks bring back more food and lose more mass than those raising female chicks [18]. Access to fewer resources in early life could increase female physiological stress levels, resulting in increased loss of telomere lengths in this sex, despite the similar lengths at the chick stage.

For thick-billed murres the sex which provides more long-term parental care is the one with slower telomere loss. This is also true in most mammalian taxa, where females provide most of the offspring care and also have longer lifespans. Males senesce quickly when there is large sexual dimorphism or high intra-sexual competition for mates [38], and long female post-reproductive lifespans have been attributed to kin selection and lower intra-sexual competition [39]. Neither of these explanations fit the monomorphic monogamous murre. In addition, female mammals have the benefits of life-extending estrogen [40] and smaller female body size effects, and again neither of these factors can explain the evidence for faster biological aging in female murres. In both mammals and murres, direct parental care by the homogametic gender (females in mammals and males in birds) is associated with slower aging [4]. However, in southern giant petrels, females invest more than males in reproduction, but still senesce more slowly [41], lending support for the idea that parental care also plays a role in driving longevity. Selection for longevity in primary care-givers could result in mechanisms maintaining male TL which are not present in females. Among the Alcidae seabird family the pattern of extended paternal care is found only in thick-billed murres and their close relatives, other bird taxa show varying male investment strategies, including complete sex-role reversal (some shorebirds) and heavy male investment (e.g. some penguins). Furthermore, sex-specific telomere loss rates have not been found in other monomorphic avian species with equal or female-biased parental care [15,27]. A phylogenetically-controlled inter-species comparison is needed to determine if the care-giving gender is longer-lived than the other and whether this trait is adaptive. Further work should attempt to link potential mechanisms to this phenomenon.

### Longitudinal Analysis of TRF Indicates Signature of Local Ecological Conditions?

Longitudinal change in TRF showed a strong colony effect. On Diabasodden, a colony with strong negative population trends [42], we see an average loss of telomeres in males (Figure 2). The other colonies did not statistically gain or lose, but trended towards slight increases in telomere length, although variability is large. Bogoslof, a colony with evidence for high food availability and increasing seabird populations [43,44], showed the most evidence for telomere length increases, and also the longest telomere lengths. St. Paul and St. George are colonies where populations are relatively stable yet nutritional stress is often high [33]; on these colonies birds maintained, but did not significantly gain or lose, TL. Based on these findings, longitudinal changes in telomeres appear to be related to colony conditions: conditions leading to low stress and positive population trends may also allow birds to elongate telomeres over a one year timeframe, but certainly do not appear to result in losses; poor conditions result in telomere deterioration, and moderate conditions lead to maintenance. Foraging conditions are linked to population trends in the Bering Sea system [45], and presumably those in the Atlantic as well, providing a demographic link between biological aging and foraging conditions. It has been previously demonstrated that early-life conditions can strongly affect telomere loss rate [25] and lifespan [46], and current habitat conditions can also affect telomere loss rates and survival in adults [47]. Telomere elongation is a more unusual finding (but see 36,48). Maintenance of telomere length over long time periods has been demonstrated in Leach’s storm-petrel [19], likely due to the actions of the telomere-lengthening enzyme telomerase [19,49], which is the likely mechanism here as well.

In conclusion, the three methods of telomere measurement all demonstrated a significant loss with age. However, TRF also indicated a sex effect: that as adults, male thick-billed murres have longer telomere lengths than females. Chicks do not differ in their lengths by sex, but as expected, have longer telomeres than adults. Thus, sex-specific patterns of telomere loss most-likely occur in the thick-billed murre between fledging and recruitment. Unlike mammals, in murres males have longer telomeres than females, and also provide the majority of parental care. Their longer telomeres may be related to their homogamous sex chromosomes or to selection for long life in the caregiving sex. Lastly, longitudinal sampling of adults on four colonies indicates that annual changes in telomere length may be related to conditions at the colony, implying that telomere change could act as a signal of physiological changes effected by ecological conditions.
