# Correction: Age, Sex, and Telomere Dynamics in a Long-Lived Seabird with Male-Biased Parental Care

**DOI:** 10.1371/annotation/d852525a-e5bc-452b-ae57-5dc2c39fed68

**Published:** 2013-09-17

**Authors:** Rebecca C. Young, Alexander S. Kitaysky, Mark F. Haussmann, Sebastien Descamps, Rachael A. Orben, Kyle H. Elliott, Anthony J. Gaston

The affiliation for the last author, Anthony J. Gaston, was incorrect.

The affiliation for this author should be: Science and Technology Branch, Environment Canada, Ottawa, ON, Canada 

